# Laser diode based THz-TDS system with 133 dB peak signal-to-noise ratio at 100 GHz

**DOI:** 10.1038/s41598-023-40634-3

**Published:** 2023-08-18

**Authors:** Vladyslav Cherniak, Tobias Kubiczek, Kevin Kolpatzeck, Jan C. Balzer

**Affiliations:** https://ror.org/04mz5ra38 grid.5718.b 0000 0001 2187 5445 NTS, University of Duisburg Essen, 47057 Duisburg, Germany

**Keywords:** Electrical and electronic engineering, Applied optics, Optical techniques, Terahertz optics, Mode-locked lasers, Ultrafast lasers

## Abstract

Terahertz time-domain spectroscopy (THz-TDS) has emerged as a powerful and versatile tool in various scientific fields. These include—among others—imaging, material characterization, and layer thickness measurements. While THz-TDS has achieved significant success in research environments, the high cost and bulky nature of most systems have hindered widespread commercialization of this technology. Two primary factors contributing to the size and cost of these systems are the laser and the optical delay unit (ODU). Consequently, our group has focused on developing THz-TDS systems based on compact monolithic mode-locked laser diodes (MLLDs). The ultra-high repetition rate (UHRR) of the MLLD has the added benefit that it allows us to utilize shorter ODUs, thereby reducing the overall cost and size of our systems. However, achieving the necessary precision in the ODU to acquire accurate terahertz time-domain signals remains a crucial aspect. To address this issue, we have developed and enhanced an interferometric extension for UHRR-THz-TDS systems. This extension is inexpensive, compact, and easy to incorporate. In this article, we present the system setup, the extension itself, and the algorithmic procedure for reconstructing the delay axis based on the interferometric reference signal. We evaluate a dataset comprising 10,000 signal traces and report a standard deviation of the measured terahertz phase at 1.6 THz as low as 3 mrad. Additionally, we demonstrate a remaining peak-to-peak jitter of only 20 fs and a record-high peak signal-to-noise ratio of 133 dB at 100 GHz after averaging. The method presented in this paper allows for simplified THz-TDS system builds, reducing bulk and cost. As a result, it further facilitates the transition of terahertz technologies from laboratory to field applications.

## Introduction

Terahertz time-domain spectroscopy (THz-TDS) using photoconductive emitters and detectors has come a long way since its inception in the late 1980s by Fattinger and Grischkowsky^1,2^. Technological and system advances have made THz-TDS a powerful and versatile tool for experimental science^3,4^. Notable milestones in improving the economy and usability of THz-TDS include the shift of the wavelength of the driving femtosecond laser to the 1.55 µm telecom band^5–7^ and the introduction of the first all-fiber spectrometer using a femtosecond fiber laser^8^. The use of improved materials and photoconductive antenna structures has made it possible to routinely achieve a bandwidth of up to 6.5 THz and the peak dynamic range of up to 111 dB^9^ with fiber-coupled systems. Recent advances in photoconductive antenna technology increased the bandwidth to 10 THz^10^. Moreover, the introduction of concepts such as asynchronous optical sampling (ASOPS)^11–13^, electronically controlled optical sampling (ECOPS)^14^, optical sampling by cavity tuning (OSCAT)^15^, and single-laser polarization-controlled optical sampling (SLAPCOPS)^16^ has made it possible to construct THz-TDS systems without a mechanical optical delay unit (ODU). Such systems tend to be more mechanically robust and—more importantly—achieve spectral update rates of up to 100.000 spectra per second^12^.

These improvements have enabled a few lighthouse applications in the field of industry. These include the characterization of graphene^17^, car paint^18^, and general non-destructive testing (NDT)^19^. A comprehensive review of the industrial applications of terahertz sensing is presented in^20^. Many more applications, including the quality control of sugar beed seeds^21^, the analysis of crude oils^22^, and quality control in the paper industry^23^, have been proven feasible but not yet managed to transition from lab demonstrations into the field. Unfortunately, the high cost of state-of-the-art THz-TDS systems still hinders their widespread deployment, and their large size and weight rule out truly mobile applications. Since the femtosecond fiber laser—despite its relative compactness—is still a major contributor to both system size and cost, there has been a lot of effort to find alternative—preferably semiconductor—light sources. In an early work just before the break of the century, Tani et al.^24^ demonstrated the generation of wide terahertz spectra by driving a photoconductive antenna with a multimode laser diode (MMLD). Subsequently, Morikawa et al.^25^ showed the use of that source in combination with a frequency-resolved power measurement for spectroscopic applications. Soon after, they made the ground-breaking discovery that a conventional time-domain spectrometer using a photoconductive emitter and a photoconductive detector generates a photocurrent that is periodic in the delay domain^26^. Since the periodicity of the photocurrent is equal to the reciprocal of the MMLD’s mode spacing, they attributed it to the cross-correlation of the fluctuating light intensity and the incident terahertz signal at the photoconductive detector, thus coining the term “terahertz cross-correlation spectroscopy” (THz-CCS). In subsequent years, this concept was improved upon by switching from a free-space optical to a fiber-coupled setup^27^ and by changing the excitation wavelength into the 1550 nm telecom band^28^. Intermittently, the concept was re-named “terahertz quasi time-domain spectroscopy” (THz-QTDS) and an improved mathematical model was developed^29^. Recently, the system bandwidth was increased by operating the MMLD with a low duty cycle^30^ and with optical feedback into the laser^31^, respectively. A variation of the THz-CCS concept using a superluminescent diode (SLD) as a mode-less semiconductor light source was first demonstrated by Molter et al.^32^ and later studied in greater detail with spectral shaping by Tybussek et al.^33^. The mode-less nature of the SLD generates a continuous terahertz spectrum, so that the system’s frequency resolution is only limited by the length of the optical delay unit (ODU). A thorough review of THz-CCS is presented in^34^.

Although the bandwidth of THz-CCS systems has quite frequently been shown to exceed 1  THz, with one work going up to 2  THz^33^, their dynamic range has been lacking compared to conventional THz-TDS systems. The highest demonstrated values range around 60 dB. This can be explained by the fact that the terahertz bandwidth is determined largely by the bandwidth of the light source, whereas the dynamic range is also affected by the infrared-to-terahertz conversion process in the photoconductive emitter and the terahertz-to-dc conversion process in the photoconductive detector. Due to the lack of a stable phase relationship between an MMLD’s laser modes, its output signal appears noise-like in the time domain. Thus, the above-mentioned processes rely on the time-averaging characteristic of the photoconductive devices, significantly lowering their efficiency. This is even more true for an SLD. Moreover, owing to these light sources’ short coherence length, the path lengths within the setup need to be accurately aligned for the infrared and the terahertz signal incident on the photoconductive detector to be correlated.

An alternative approach that was first demonstrated by Merghem et al.^35^ in 2017 is the use of a monolithic mode-locked laser diode (MLLD). Although the bandwidth and dynamic range achieved with this approach was rather modest at first, the switch from a conventional photoconductive emitter to an antenna-integrated photodiode led to an increase of the bandwidth and of the peak dynamic range to about 1.5 THz^36^ and 70 dB^37^, respectively. While early works regarded this approach as a variation of THz-CCS/QTDS, a detailed system-theoretical analysis revealed that the long coherence length of the MLLD makes this concept much more akin to “conventional” THz-TDS^36^. The analysis revealed the exact relationship between the complex laser spectrum and the detected terahertz spectrum. Most importantly, it was found that the highest terahertz spectral amplitudes are realized in the case of a linear phase relationship between the laser modes, i.e. in the case of unchirped laser pulses. Paying tribute to the pulse repetition rate of several 10 GHz of the MLLD, this concept was later coined “ultra-high repetition rate terahertz time-domain spectroscopy” (UHRR-THz-TDS)^38^. Typical values range around 50 GHz, resulting in a detected photocurrent with a period of around 20 ps. This has two practical advantages. Firstly, the ODU needs only cover a delay range of 20 ps. Secondly, there is no need to temporally align the infrared and terahertz pulses at the photoconductive detector. The short ODU can enable a compact system and a faster acquisition rate. The use of a fast—possibly free-running—ODU, requires the generation of a reference signal for accurate reconstruction of the delay axis. It was shown by Molter et al.^39^ that such a reference signal can be generated in a THz-TDS system by constructing a free-space optical Mach-Zehnder interferometer around the ODU and polarization multiplexing the infrared signal of an additional continuous-wave laser through that interferometer. We later demonstrated that this approach can be adapted in a simplified way in a fully fiber-coupled UHRR-THz-TDS system^38^. Because of the MLLD’s high repetition rate and comparatively small bandwidth, we could use it to drive both the terahertz spectrometer and the interferometer, thus eliminating the need for an additional continuous-wave laser and polarization multiplexing. By only considering the envelope of the interferogram, we were able to synchronize terahertz traces with a time accuracy of a few femtoseconds. However, the dynamic range still fell off compared to the state-of-the-art.

**Figure 1:**
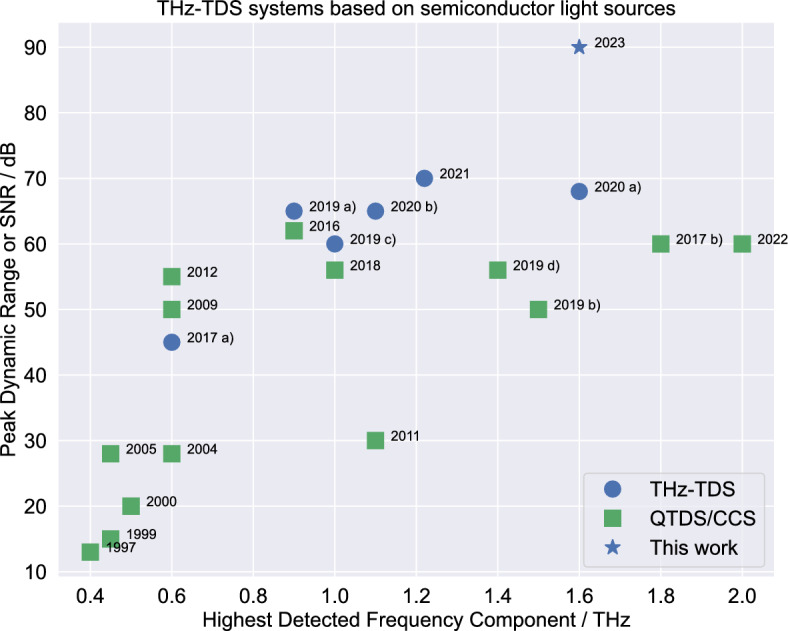
Development of the peak dynamic range or SNR and of the detected bandwidth of THz-TDS systems based on monolithically integrated light sources. Since the quantities dynamic range and SNR are used somewhat ambiguously in the analyzed literature, the two terms are used alike in this chart. Green colored squares represent QTDS/CSS based systems. Blue colored circles represent THz-TDS systems. The star shaped marker puts this work into perspective. The individual references can be found in Table 1.

In this work, we extend this concept by using the envelope of the interferogram for synchronization and the interferogram itself for correction of the delay axis. We demonstrate an UHRR-THz-TDS system with a bandwidth of 1.6 THz and a record-high single-shot peak signal-to-noise power ratio (SNR) of 90 dB at 100 GHz. These values are placed into the context of the development of THz-TDS systems based on monolithic light sources by the chart shown in Fig. 1 and the respective Table 1. It should be noted that we specify peak SNR, whereas some other publications specify peak dynamic range. The process by which we determine the SNR is described in the section “Signal stability and validity evaluation”. There are three main innovations contributing to achieving the record-high SNR:

- We perfectly compensate the pulse chirp of the MLLD with a section of single-mode fiber and a programmable optical filter (POF).
- We synchronize and correct the delay axis of the detected photocurrent using a reference signal generated with the above-mentioned interferometric approach. This enables us to highly accurately average 10,000 pulses for maximizing the SNR without employing an additional laser.
- We use optimized algorithms for processing the measured data, thereby taking into account and profitting from the periodicity of the measured signals.

This paper is structured as follows. First, we present a detailed description of our measurement setup, followed by the respective mathematical representation of the interferometric monitoring aspect. Further, we present measurements that validate the mathematical model. Next, we present step-by-step algorithmic processing of the interferometric signal to calculate the delay axis. This algorithm is used to process a large set of terahertz measurements. Using the processed data set, we investigate the averaging performance, system jitter, and phase stability of the post-processed data. For further verification, we compare the measurement results with a system theoretical model.

## Results

### System configuration for interferometric optical delay line observation with ultra-high repetition rate laser diodes

**Figure 2:**
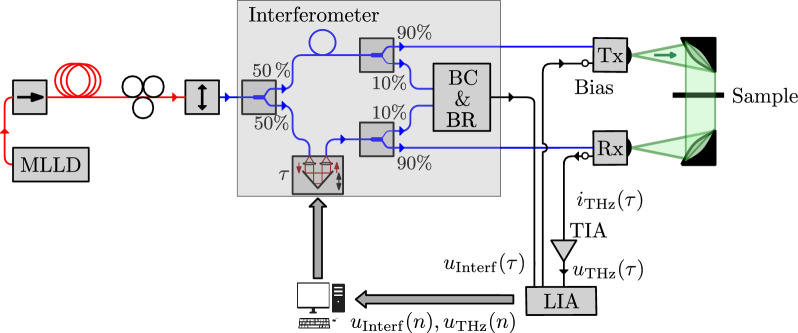
Standard THz-TDS setup driven by an MLLD and extended by two additional optical splitters, an optical combiner, and a BR to generate an additional interferometric signal for ODU position monitoring during measurements. Single-mode fibers, polarization-maintaining fibers, and electrical connections are depicted by red, blue, and black colored lines, respectively.

**Figure 3:**
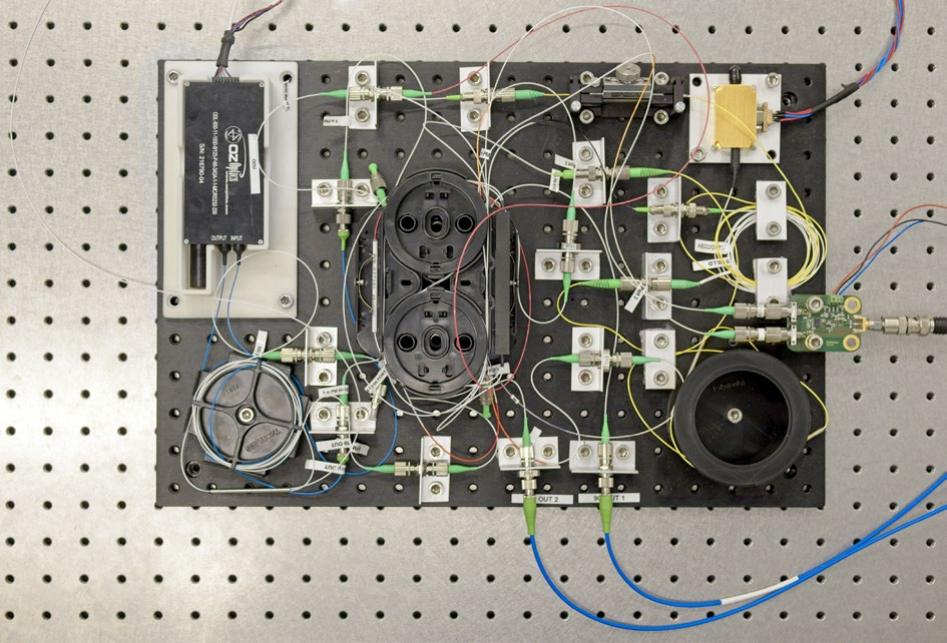
Photograph of the UHRR-THz-TDS system constructed on a 45 cm by 30 cm aluminum breadboard. Key components are the MLLD at the top right corner, the ODU in the top left corner, the 50 m long fiber spool at the bottom right corner, the balanced receiver at the right edge, and the polarization controller to the left of the MLLD. Most of the other fiber optic components are stacked in the middle of the breadboard. The terahertz transmitter and receiver are connected via the blue PM fibers at the bottom edge. Not visible in the photograph is standard lab equipment used in the setup, i.e., the laser driver, the EDFA, the POF, the OSA, the autocorrelator, the TIA, the LIA, and the lab personal computer.

The measurement setup as depicted in Fig. 2 illustrates a commonly used THz-TDS setup supplemented by the components for the interferometer and a monolithically integrated MLLD. The laser light emitted by the MLLD is fed through an optical isolator to prevent feedback, 50 m of standard single-mode fiber for pulse compression, a polarization controller, and a polarizer. In addition, a programmable optical filter (POF, Finisar WaveShaper 1000A) is used to fine-tune the pulse compression. An erbium-doped fiber amplifier (EDFA, Thorlabs EDFA100P) amplifies the optical signal to 30 mW per antenna after the POF for optimal experiment conditions. An optical beam splitter with a 50/50 ratio feeds the signal into an optical delay unit (ODU, ODL-650) and a 99/1 splitter. We use 1% of the optical power to measure the optical spectrum using an optical spectrum analyzer (OSA, Anritsu MS9740A). At this point, both optical arms are usually routed to the terahertz antennas after the ODU and the 99% splitter. Instead, we replicated the interferometric setup proposed by Kolpatzeck et al.^38^. We add two 90/10 splitters in each arm. Both 10% outputs are combined by a 50/50 coupler and feed a balanced-receiver (BR). The 90% outputs feed both antennas or an autocorrelator (APE PulseCheck) to measure the optical pulse width at the antennas. The transmitter (Tx, Toptica #EK-000724) is a biased antenna-integrated photodiode, whereas the receiver (Rx, Toptica #EK-000725) is an unbiased photoconductive antenna. For lock-in detection, the transmitter bias is modulated by the lock-in amplifier’s (LIA, Zurich Instruments MFLI Lock-in Amplifier) reference signal from $-1.4$ to 0.5 V. The detected photocurrent from the terahertz receiver is amplified by a transimpedance amplifier (TIA, Femto HCA-4M-500K) and measured with the LIA. In parallel, the output of the balanced receiver (BR) is also measured by the LIA using an auxiliary input. With this setup, we are able to measure the optical spectrum, autocorrelation function, interferometer signal, and terahertz signal. For optimal alignment and to obtain the antenna and path characteristics we perform a continuous wave (CW) terahertz measurement using a commercial terahertz frequency-domain spectroscopy (THz-FDS) system (Toptica TeraScan 1550). Furthermore, we use the optical spectrum to calculate the optimal autocorrelation functions assuming chirp-free pulses. The calculation yields a theoretically achievable optical pulse width of 585 fs. Based on this result we fine-tune the dispersion compensation utilizing the POF, achieving an autocorrelation width of 640 fs. Considering the work of Ding et al.^40^, we find a high-quality pulse compression within our measurement setup. The remaining two measurements are the terahertz photocurrent and the interferometer signal at the BR output. A photograph of the laboratory realization is shown in Fig. 3.

### Theoretical calculation of the balanced-receiver output

**Figure 4:**
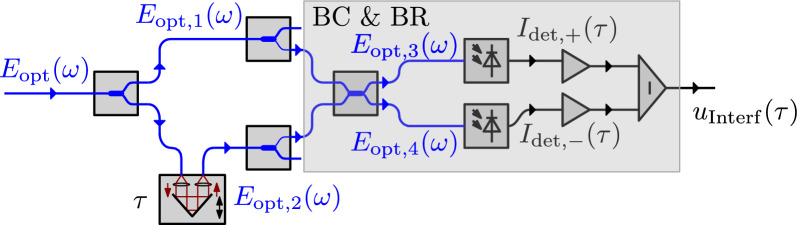
Detailed representation of the interferometer from Fig. 2, including labels of the optical and electrical signal flow.

We utilize the interferometer signal to synchronize subsequent terahertz measurements and to calculate their respective time axes. Therefore, we partly reintroduce the formulations from Kolpatzeck et al.^38^. However, here we chose a frequency-domain representation to allow for a more compact matrix representation of the signal path to the BR. To aid the formulations, Fig. 4 depicts the interferometer as an extract of the measurement setup, including the beam combiner (BC) and BR.

The complex optical spectrum of an MLLD can be approximated as a function of the angular frequency $\omega$ by the following expression:

1 $$\begin{aligned} E_\text{opt}(\omega ) {\propto} {\sum}_{k=0}^{N-1} E_k (\delta [\omega + (\omega _0+k\Delta \omega )]{\text{e}}^{{-\mathrm j} {\phi}_{\text{k}}} + \delta [\omega - (\omega_0+k\Delta \omega )] {\text{e}}^{\text{j}{\phi}_{\text{k}}} ), k \in {\mathbb{N}} \end{aligned}$$

where *k* is the numbered index of each frequency component within the optical spectrum starting at 0. $E_k$ and $\phi _k$ describe the respective amplitudes and phases. Further, $\Delta \omega$ and $\omega _0$ are equal to $2 \pi F$ and $2\pi f_0$, respectively, with *F* being the free spectral range (FSR) and $f_0$ being the first frequency component of the optical spectrum. Simplifying expression (1) by assuming equal phases with $\phi _k = 0$ yields

2 $$\begin{aligned} E_\text{opt}(\omega ) \propto \sum _{k=0}^{N-1} E_k (\delta [\omega + (\omega _0+k\Delta \omega )] + \delta [\omega - (\omega _0+k\Delta \omega )] ). \end{aligned}$$

Now the transfer matrix of an optical fibre coupler can be written as

3 $$\begin{aligned} {\textbf{A} }=\frac{1}{\sqrt{2}} \begin{pmatrix} 1 &{} \text{j} \\ \text{j} &{} 1 \end{pmatrix}. \end{aligned}$$

Evaluating the coupler paths up to $E_\text{opt,3}(\omega )$ and $E_\text{opt,4}(\omega )$ results in the input vector for the last coupler

4 $$\begin{aligned} \textbf{E} = \left( \begin{array} {c} \text{j} \textit{E}_{\text{opt}}(\omega ) \\ -E_\text{opt}(\omega )\text{e}^{\mathrm{-j}\omega \tau } \end{array}\right) \end{aligned}$$

leading to an input vector for the BR with

5 $$\begin{aligned} {\textbf{A} } \cdot \left( \begin{array} {c} E_\text{opt,3}(\omega ) \\ E_\text{opt,4}(\omega ) \end{array}\right) \propto \begin{pmatrix} 1 &{} \text{j} \\ \text{j} &{} 1 \end{pmatrix}\cdot \left( \begin{array} {c} \text{j} \textit{E}_\text{opt}(\omega ) \\ -E_\text{opt}(\omega )\text{e}^{\mathrm{-j}\omega \tau } \end{array}\right) = \left( \begin{array} {c} \text{j}{} \textit{E}_\text{opt}(\omega ) - \text{j}{} \textit{E}_\text{opt}(\omega )\text{e}^{\mathrm{-j}\omega \tau } \\ -E_\text{opt} (\omega )- E_\text{opt}(\omega )\text{e}^{\mathrm{-j}\omega \tau } \end{array}\right) = \left( \begin{array} {c} \text{j} \textit{E}_\text{opt}(\omega )(1 - \text{e}^{\mathrm{-j}\omega \tau }) \\ -E_\text{opt} (\omega )(1+ \text{e}^{\mathrm{-j}\omega \tau } ) \end{array}\right) . \end{aligned}$$

Evaluating Eq. (2) and the first component of (5) leads to

6 $$\begin{aligned} E_\text{opt, 3}(\omega ) \propto \sum _{k=0}^{N-1} E_k (\delta [\omega + (\omega _0+k\Delta \omega )](1 - \text{e}^{\mathrm{-j}(\omega _0+k\Delta \omega )\tau }) + \delta [\omega - (\omega _0+k\Delta \omega )](1 - \text{e}^{\text{j}(\omega _0+k\Delta \omega )\tau }) ). \end{aligned}$$

The photocurrent detected by the individual inputs of the BR is proportional to the average optical power of the incident wave.

7 $$\begin{aligned} I_{\text{det},+}(\tau ) = \mathscr {F}^{-1}\{[E_\text{opt,3}(\omega )*E_\text{opt,3}(\omega )] \cdot H_\text{LP}(\omega )\}, \end{aligned}$$

where $H_\text{LP}(\omega )$ is the transfer function of a low-pass system that cuts off frequency components much higher than *F*. This results in

8 $$\begin{aligned} I_\mathrm{det,+}(\tau ) = \sum _{k=0}^N E_k^2 [1-\cos (\{\omega _0+k\Delta \omega \}\tau )] \end{aligned}$$

and analog

9 $$\begin{aligned} I_{\text{det},-}(\tau ) = \sum _{k=0}^N E_k^2 [1+\cos (\{\omega _0+k\Delta \omega \}\tau )]. \end{aligned}$$

Finally, the output of the BR is proportional to the difference between both detected photocurrents, which gives

10 $$\begin{aligned} I_\text{det}(\tau ) \propto I_{\text{det},-}(\tau )-I_{\text{det},+}(\tau ) = \sum _{k=0}^N E_k^2 \cos ([\omega _0+k\Delta \omega ]\tau ). \end{aligned}$$

**Figure 5:**
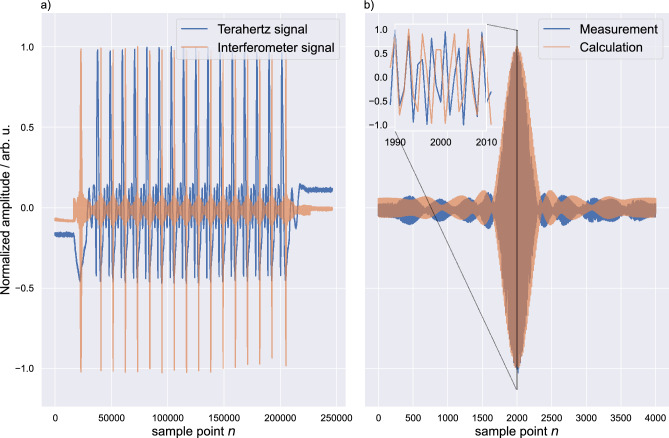
In (**a**) we show raw measurement data of the terahertz and interferometer signal in blue and orange respectively. Both signals are measured in parallel using the LIA. While the terahertz signal is measured via the implemented lock-in procedure, the interferometer signal is measured using a standard auxiliary port. A magnification of the interferometer signal in blue is depicted in (**b**) and compared with the theoretical solution calculated with Eq. 10 in orange. An additional inset shows a further magnification, highlighting the high-frequency fringes of the interferometer signal.

In Fig. 5a, we show an exemplary measurement of the terahertz signal in blue as well as the interferometric signal in orange. In Fig. 5b, we compare the measured interferometric signal in blue to the waveform calculated from Eq. 10 in orange. A key difference of the here-used MLLD compared to standard CW optical interferometer solutions is the ultra-high repetition rate of 50 GHz. This lead to the periodic terahertz time domain signal with a periodicity of 20 ps corresponding to 1/*F*. Over the ODUs length with a maximum time delay of 330 ps, we hence measure a total of 16 terahertz pulses including the acceleration and declaration phase of the ODU. The same periodicity can be seen in the interferometer signal. Additionally, high-frequency fringes are measured within the interferometer signal. The frequency of these can be approximated as $f_0$ and corresponds to the first frequency component of the optical spectrum. The only information missing at this point is a time axis. To acquire this information we use several characteristics of the interferometer signal and explain the procedure in the signal processing section.

### Signal processing

**Figure 6:**
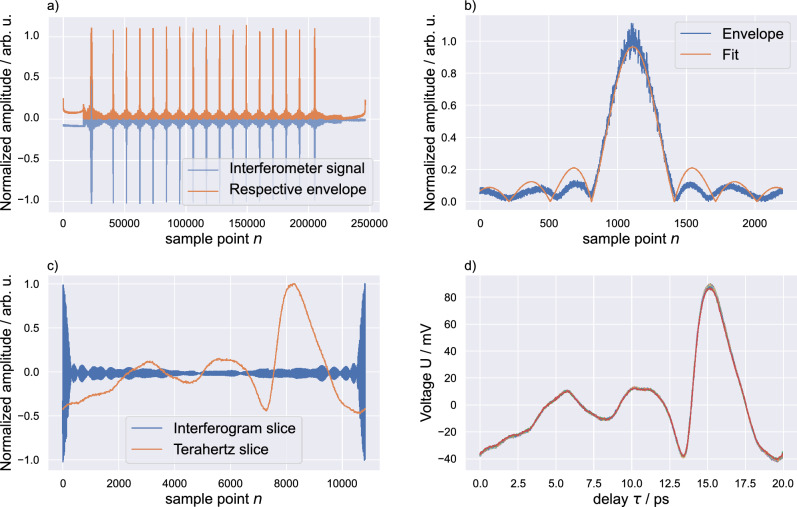
(**a**) Raw measurement data of the interferometer signal and the respective envelope. Both signals are measured in parallel using the LIA. While the terahertz signal is measured via the implemented lock-in procedure, the interferometer signal is measured using a standard auxiliary port on the measurement device. An exemplary fit for an individual slice of the interferograms envelope is depicted in (**b**). Based on the subsequent slices found by fitting all envelopes, we get the corresponding slice for the terahertz signal as shown in (**c**). (**d**) All terahertz pulses measured within a single measurement and sliced by the here-described algorithm.

The goal of the signal processing is to extract all individual terahertz pulses as well as calculate a highly precise time axis for each pulse while maintaining phase relations between subsequent measurements. In our measurement setup, the terahertz signal is influenced by the free space antenna setup. Whereas, the interferometer signal comes from a fully fiber-coupled setup and depends only on the light source, ODU, and temperature. The interferometric signal contains two pieces of relevant information. First, the distance between the local maxima of its envelope is the reciprocal repetition period of the MLLD. Therefore, the envelope of the signal can be used to trigger the acquisition and slice individual terahertz pulses. Second, Eq. 10 shows that the high-frequency fringes can be approximated with $\omega _0$. Hence, we can use their local extrema for precise estimation of the ODU’s position during measurements. With these two features, we use the interferometer signal to find the right positions for slicing the data and for calculating the precise time axis. The algorithm used to process the raw terahertz measurements and raw interferometer signals is described in the following steps, as well as elaborated step by step in Fig. 6.

- We start by calculating the envelope of the interferometer signal, as shown in Fig. 6a. Here, we use the absolute of a Hilbert-transform applied to the raw interferometer signal.
- Next, we want to find points at which we can slice the data based on the envelope. We use the periodicity of the signal and search for the maximum of each period. Due to the square-like shape of the optical spectrum, the interferometer signal and its envelope resemble a sinc function, as depicted in Fig. 6b. Therefore, we implement a curve-fitting algorithm for the envelope:
  11 $$\begin{aligned} f(x) = \left| {A\cdot \text{sinc}\left( {\frac{x-x_0}{B}}\right) }\right| \end{aligned}$$
  where *A* and *B* are scaling factors and $x_0$ is the offset of the envelope. Hence, $x_0$ is used to determine the exact position of each period.
- We slice both the raw interferometer signal and the raw terahertz signal, resulting in individual pulses as seen in Fig. 6c. Note that no time axis is constructed yet.
- To calculate a time axis we use the a priori information given by the high-frequency fringes of the interferometer signal. From Eq. 10, we see that the frequency of the fringes is $\mathrm f_0$. Here we make the assumption that $\mathrm f_0$ is the first frequency component of the optical spectrum, which is $\mathrm f_0 \approx$ 192 THz.We calculate all minima and maxima of the high-frequency fringes within the sliced interferometer signals by performing a zero-crossing search on the signal’s derivative. Based on the found minima and maxima the sliced terahertz signals are resampled. This leads to a reduction in data points and all sample points of the terahertz signal are now equidistant with a time step of half the fringe period, giving us a time axis fully based on the interferometer signal. The final terahertz signal is depicted in Fig. 6d, giving us single signal periods with a duration of approximately 20 ps.

Within the ODU’s maximum delay of 330 ps we can measure up to 16 individual terahertz pulses. We remove the first and last pulse measured during the acceleration and deceleration phases of the ODU, where strong non-linearity of the time-axis occurs. This yields 14 terahertz pulses per measurement for further analysis and verification of the described algorithm.

### Signal stability and validity evaluation

**Figure 7:**
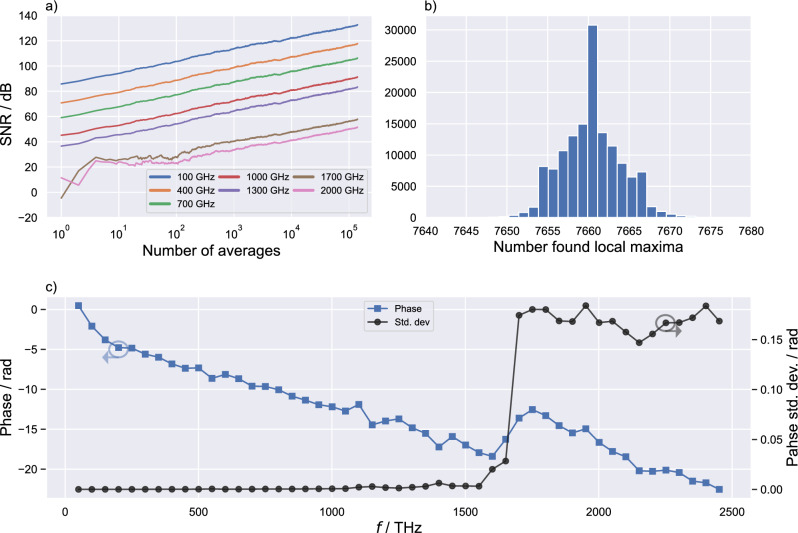
(**a**) Increase in SNR with a rising number of averages for individual frequency components, with the highest SNR begin 133 dB at 100 GHz . (**b**) Histogram of the number of found local maxima within individual slices, with a std. dev. of approx. 3.6. (**c**) Frequency resolved phase of an average with 140,000 individual terahertz pulses and phase std. dev. calculated from averaging groups of 10.

To verify the validity of the presented method, we analyze the acquired data from a measurement with 10.000 subsequently measured terahertz traces. Each trace includes 14 individual terahertz pulses. With the here-used ODU we achieve a measurement time of 2.4 seconds per trace, resulting in a total measurement time of approximately 6.5 h. Important to note here is that the measurement time is primarily determined by the travel speed of the ODU. The noise level is determined by conducting the same measurement with a thick metal plate blocking the line-of-sight connection between the antennas. As the interferometer is not reliant on the terahertz path, it remains usable for slicing and determining the time axis, similar to its application in terahertz measurements. Therefore, we average the same number of measurements as in the terahertz measurement and calculate the noise level from this data set. Using this data set, we show in Fig. 7 three different aspects to evaluate the performance of the presented method. First, we analyze the development of the SNR with an increasing number of averages. As depicted in the double logarithmic plot presented in Fig. 7a a linear increase of the SNR for frequencies up to 1300 GHz can be seen. Due to the low-pass characteristic of the antennas components above 1300 GHz are not detectable from a single measurement. Only with a precisely reconstructed time axis and averaging the SNR can be improved sufficiently to enable detection. The linear increase of the SNR in a double logarithmic plot is in good agreement with the well-known $\sqrt{n}$ rule for averaging. Starting with a peak SNR of approx. 85.5 dB at 100 GHz, we achieve a peak SNR of 132.7 dB with averaging. With overall 140,000 terahertz pulses, we achieve an increase in peak SNR of 47.2 dB, which is close to the theoretically achievable increase is 51.4 dB.The next metric we use is the number of found maxima within a slice. Ideally, each trace should have the same length and period. However, due to uncertainties within the peak finding a small peak-to-peak jitter remains. In Fig. 7b we show a histogram of the number of maxima found for each sliced terahertz pulse. The performance of our algorithm is validated by a standard deviation of 3.6 found maxima, resulting in a remanent peak-to-peak jitter of approx. 20 fs. This is comparable with the results of other groups utilizing interferometric monitoring employing an additional laser^41^.Last, we analyze the phase development and uncertainties of our dataset. To be able to analyze the phase stability at higher frequencies, averaging has to be done to increase the SNR. However, to keep the sample size high for statistical analyses we chose to randomly group 10 pulses for averaging. Resulting in 14,000 individual, 10 times averaged terahertz pulses. Using this new dataset we calculate the average phase and standard deviation as a function of the frequency as shown in Fig. 7c. Due to the terahertz antenna’s low-pass characteristic and propagation through air, we measure a linearly decreasing phase up to 1600 GHz, at which the standard deviation starts to increase. This indicates a high phase stability and averaging performance up to 1600 GHz, well fitting to the SNR development results in Fig. 7a. The standard deviation of the phase at 1.6 THz is as low as 0.003 rad.

### Performance improvement

**Figure 8:**
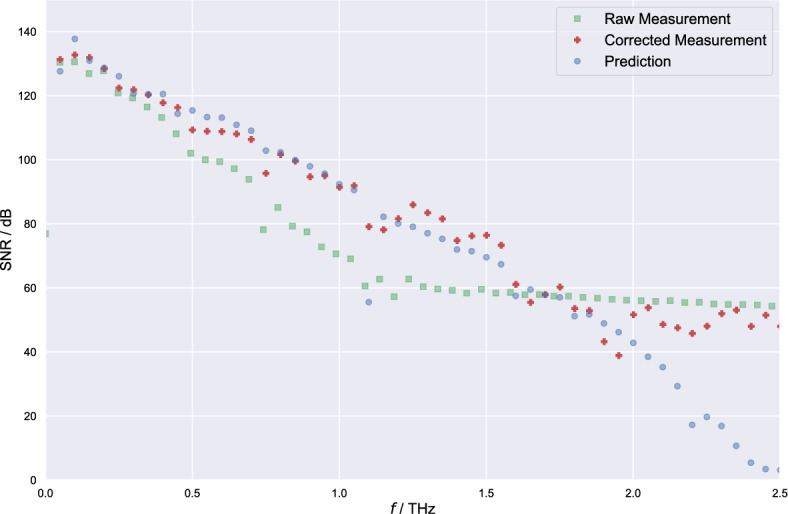
Frequency domain representation of 140,000 averaged single terahertz pulses, with and without correction, compared to a theoretical model-based prediction, achieving an SNR of 133 dB.

First of all, we use our system theoretical model to compare our measurement results with a calculated terahertz spectrum. The calculation is performed by using the before mentioned MLLDs measured optical spectrum as well as the terahertz paths transfer function measured by a THz-FDS system. The model yields a terahertz spectrum by calculating an ideal terahertz pulse generated by the MLLD at ideal pulse compression convoluted with the impulse response of the spectrometer. The result of the calculation does not yield an assumption about the contained power. Therefore, we normalize and scale the amplitude result according to our measurements. We then compare the shape and evolution of the prediction with respect to our measurement result, before and after applying the interferometric correction. Fig. 8 depicts a good match between calculation and measurement. The interferometric correction improves the evolution of the signal towards higher frequencies, ultimately achieving a good match between calculation and measurement results. The overall average of the here used 140.000 individual terahertz pulses yields a peak SNR of 133 dB.

## Discussion

The interferometric observation of ODUs as presented in this work provide both, a method for synchronizing subsequent measurements and calculating a highly precise time axis for optical sampling-based terahertz applications. The algorithmic extension and improvements towards the measurement system yield a remaining peak-to-peak jitter as low as 20 fs, competing with similar state-of-the-art solutions^41^. Further, we show phase instabilities as low as 0.003 rad up to 1.6 THz and a record-high peak SNR of 133 dB compared to previous publications^9^ as a result of improved averaging performance for subsequent measurements. All in all the interferometer extension consists of three additional fiber-couplers, a BR, and a second data acquisition channel. Hence, no additional laser sources or otherwise expensively monitored ODU are required, all while drastically increasing the averaging performance and phase stability of subsequent measurements. As a very simple and inexpensive extension, this measurement method can improve several existing THz-TDS systems, even at lower repetition frequencies.

## Methods

### Laser diodes

The MLLDs used in this work are InAs/InP QD and InGaAsP/InP QW introduced by Zander et al.^42^. In a previously published work^43^ we have investigated the stability of these MLLDs at different points of operations. Based on this work, we operate the MLLDs at optimal points of operation with respect to their repetition rate stability.

### Literature research

Research on THz-systems based on monolithically integrated light sources was conducted and depicted in Fig. 1. Additionally, a tabular representation containing the cross references is provided here in Table 1.

**Table 1:** Authors including references, publication year and system type respective to Fig. 1. Publications denoted with an “*” do not provide a clear value for dynamic range and/or bandwidth. These are approximations extracted from published figures.

Author	Year of publication	System type	Peak dynamic range or SNR/dB	Bandwidth/THz
* M. Tani et al.^24^	1997	QTDS/CCS	13	0.4
O. Morikawa et al.^25^	1999	QTDS/CCS	15	0.45
O. Morikawa et al.^26^	2000	QTDS/CCS	20	0.5
O. Morikawa et al.^27^	2004	QTDS/CCS	28	0.6
M. Tani et al.^44^	2005	QTDS/CCS	28	0.45
M. Scheller et al.^29^	2009	QTDS/CCS	50	0.6
D. Molter et al.^45^	2011	QTDS/CCS	30	1.1
M. Scheller et al.^46^	2012	QTDS/CCS	55	0.6
J. C. Balzer et al.^47^	2016	QTDS/CCS	62	0.9
K. Merghem et al.^35^	2017a	TDS	45	0.6
R. Kohlhaas et al.^28^	2017b	QTDS/CCS	60	1.8
A. Rehn et al.^30^	2018	QTDS/CCS	56	1
J. C. Balzer et al.^48^	2019a	TDS	65	0.9
D. Molter et al.^32^	2019b	QTDS/CCS	50	1.5
S. Tonder et al.^49^	2019c	TDS	60	1
K. Tybussek et al.^50^	2019d	TDS	56	1.4
K. Kolpatzeck et al.^36^	2020a	TDS	68	1.6
V. Cherniak et al.^51^	2020b	TDS	65	1.1
V. Cherniak et al.^37^	2021a	TDS	70	1.22
K. Tybussek et al.^33^	2022	QTDS/CCS	60	2

## Data Availability

The data that support the findings of this study are available from the authors on reasonable request.
